# Checkpoint Inhibition in Myeloma: Opportunities and Challenges

**DOI:** 10.3389/fimmu.2018.02204

**Published:** 2018-09-26

**Authors:** Federica Costa, Rituparna Das, Jithendra Kini Bailur, Kavita Dhodapkar, Madhav V. Dhodapkar

**Affiliations:** ^1^Department of Medicine and Surgery, University of Parma, Parma, Italy; ^2^Winship Cancer Institute, Emory University, Atlanta, GA, United States

**Keywords:** myeloma, immunotherapy, immune checkpoint, PD-1, PD-L1

## Abstract

Despite major improvements in the treatment landscape, most multiple myeloma (MM) patients eventually succumb to the underlying malignancy. Immunotherapy represents an attractive strategy to achieve durable remissions due to its specificity and capacity for long term memory. Activation of immune cells is controlled by a balance of agonistic and inhibitory signals via surface and intracellular receptors. Blockade of such inhibitory immune receptors (termed as “immune checkpoints”) including PD-1/PD-L1 has led to impressive tumor regressions in several cancers. Preclinical studies suggest that these immune checkpoints may also play a role in regulating tumor immunity in MM. Indeed, myeloma was among the first tumors wherein therapeutic efficacy of blockade of PD-1 axis was demonstrated in preclinical models. Expression of PD-L1 on tumor and immune cells also correlates with the risk of malignant transformation. However, early clinical studies of single agent PD-1 blockade have not led to meaningful tumor regressions. Immune modulatory drugs (IMiDs) are now the mainstay of most MM therapies. Interestingly, the mechanism of immune activation by IMiDs also involves release of inhibitory checkpoints, such as Ikaros-mediated suppression of IL-2. Combination of PD-1 targeted agents with IMiDs led to promising clinical activity, including objective responses in some patients refractory to IMiD therapy. However, some of these studies were transiently halted in 2017 due to concern for a possible safety signal with IMiD-PD1 combination. The capacity of the immune system to control MM has been further reinforced by recent success of adoptive cell therapies, such as T cells redirected by chimeric-antigen receptors (CAR-Ts). There remains an unmet need to better understand the immunologic effects of checkpoint blockade, delineate mechanisms of resistance to these therapies and identify optimal combination of agonistic signaling, checkpoint inhibitors as well as other therapies including CAR-Ts, to realize the potential of the immune system to control and prevent MM.

## Immune system as an effective approach to treat cancer and principles of immune checkpoint blockade

The role of immune system in cancer progression has been studied for over a century (1). However, only recently immunotherapy has emerged as an effective strategy to treat several types of cancers with impressive results in terms of tumor regression and durable remissions (2). The concept of immune surveillance and editing of tumors is now well-accepted (3).

Several studies suggest a role for genetic and epigenetic modifications in cancer development and progression (4–6) and some of them correlate with the ability to escape this immunosurveillance (5, 6). Tumor cells can indeed lower their immunogenicity through the down regulation of MHC-mediated neo-antigen presentation, accompanied by deletion of cancer cells expressing T cell targets (immunoediting) (3). The immunoediting process in cancer pathogenesis comprises of three phases: elimination, equilibrium and escape. In the first phase, the innate and adaptive immune systems recognize and eradicate cancer cells through the cytolytic activity of immune cells (i.e., NK cells, NKT cells, γδ T cells, and CD8^+^ T cells), antibody-dependent cell-mediated cytotoxicity (ADCC), or complement-dependent cytotoxicity (CDC) mechanisms (7, 8). In the equilibrium phase, a balance between cancer progression and cancer elimination is established through the modulation of control checkpoints (3, 7). However, if cancer persists, it overcomes the immunity response and escapes with further progression and metastasis (3, 7).

Along with the suppression of tumor antigen expression, different mechanisms that involve surface molecules and soluble factors released in the tumor microenvironment, e.g., indoleamine 2,3-dioxygenase (IDO), type I interferons (IFNs) and IFN-γ, galectin-1, have been described in the disruption of immune homeostasis and in the altered balance from effector to regulatory and suppressive cells induced by cancer (7, 9).

In principle, immunotherapy could either enhance the immune response or inhibit tumor suppression (10). The most commonly used approach is the modulation of inhibitory immune receptors (termed as “immune checkpoints”) that regulate the balance between immune response and immune tolerance (11). Several studies showed that cancer cells increase the expression of some checkpoint proteins (summarized in Table 1), such as programmed cell death ligand-1 (PD-L1), with inhibitory properties on T cell functions, as a mechanism of immune resistance (20). These results lead to the development of monoclonal antibodies (mAbs) directed against such immune checkpoints, further approved for the treatment of several solid tumors as melanoma, renal and lung cancer (21–23).

**Table 1:** Immune checkpoint distribution and functions.

**Checkpoint**	**Expression**	**Function**	**References**
CTLA4 (CD80/CD86)	Activated T cells and Tregs	Inhibition of CD28 co-stimulation and T cell activity; Enhancement of Treg functions; Induction of TGF-β and IDO	(12) (13)
PD-1 (PD-L1/PD-L2)	Activated T cells, NK cells, NKT cells, B cells, Monocytes, DCs, MDSCs	T cell exhaustion and apoptosis; inhibition of cytokine production; downregulation of NK and NKT cell activity	(14)
LAG3 (MHCII)	activated T cells, NK cells, B cells, pDCs	Effector T cell inhibition; Increased Treg activity	(15)
TIM3 (Galectin 9, HMGB1)	Exhausted T cells, NK cells, NKT cells, B cells, DCs, Macrophages	Th1 cell apoptosis; Reduced cytokine release; Induction of tolerogenic M2 phenotype	(16) (17)
TIGIT (CD155)	Exhausted cytotoxic T cells, NK cells	Effector T cell inhibition; Reduced NK cell cytotoxicity; Enhanced Treg activity	(18) (19)

*DCs, dendritic cells; MDSCs, myeloid derived suppressor cells; NK, natural killer; NKT, NK-like T cells; Th, T helper; Tregs, T regulatory cells*.

CTLA-4 is the first immune-checkpoint explored as a clinical target (24). It is normally expressed at low levels on the surface of effector T cells and regulatory T cells (Tregs) and it is involved in the early stages of T cell activation (25). CTLA-4 shares the same ligands of CD28 (CD80 and CD86) expressed on antigen presenting cells (APCs). Once CD28 binds CD80 or CD86 to provide co-stimulation, the inhibitory CTLA-4 molecule is shuttled to the T cell surface where it binds CD80 or CD86 with higher affinity (26) thus counteracting the costimulatory activity of CD28 through the binding of the phosphatases PP2A and SHP-2 (25, 27). CTLA-4 expression also exerts its immunosuppressive functions by other mechanisms, including Treg expansion and induction of immunosuppressive cytokines, such as transforming growth factor (TGF)-β and the enzyme IDO (13, 21). While CTLA4 expression is mostly studied for its expression on lymphoid cells, recent studies suggest that myeloid dendritic cells can secrete CTLA4^+^ microvesicles that may mediate immune suppression (28). CTLA-4 blockade with mAbs (i.e., ipilimumab) can then enhance the immune response against tumor by inactivating Treg, tumor-infiltrating lymphocytes (TILs) (29) and increasing T helper (Th)1 cell functions (20).

PD-1 is a member of the CD28/CTLA-4 family, with inhibitory properties, mainly expressed on exhausted T cells (dysfunctional T cells classically associated with chronic infection), NK and NKT cells following activation (14). APCs, monocytes and malignant cells express its ligands, PD-L1 and PD-L2, especially under inflammatory conditions (14).

Similarly to CTLA-4, the interaction between PD-1 and PD-L1 interferes with TCR signal transduction, by recruiting the tyrosine phosphatase SHP-2 and subsequent inactivating the PI3 kinase-signaling cascade (30, 31), which leads to reduced cytokine synthesis, cytotoxic functions and blockade of T cell proliferation and survival (14).

In the physiologic setting, this pathway enables the immunologic equilibrium after initial T cell response, preventing over-activation and the possible expansion of auto-reactive T cells (32). Studies on PD-L1^−/−^ murine models reported an accumulation of effector T cells along with an increased IFN-γ production by CD8^+^ T cells, suggesting an impaired apoptosis regulation in the absence of PD-L1 (33). Moreover, spontaneous accumulation of CD8^+^ T cells occurred in the liver even in the absence of “non-self” antigen exposure leading to the development of multiple autoimmune features (33). These data highlighted the importance of PD-L1 in controlling the responses of self-reactive T cells that have escaped into the periphery. In addition, PD-1/PD-L1 axis regulates the dynamic interplay between Tregs and T effector cells. In the presence of inflammatory milieu, PD-L1 expressed on both APC and naïve Tregs induces PD-1 expression on naïve T cells and promotes their differentiation toward a regulatory phenotype and function (induced Tregs) (34). On the other hand, a negative feedback loop occurs to downregulate Treg development and function. This effect is mediated by the increased PTEN expression via PD-1 signaling which in turn reduces PD-L1 expression on Tregs (35). Finally, PD-L1^+^ Tregs directly induce a tolerogenic phenotype in APCs that reduces the priming of T effector cells (34). All these results thus confirmed the critical role of the PD-1/PD-L1 pathway in the balance between T cell activation and tolerance.

According to the relevance of PD-1/PD-L1 axis in immune control, tumors seem to highjack this pathway to suppress and escape the activation of an immune response (36). High PD-L1 expression is associated with a poor prognosis in solid tumors, including lung, ovarian or colon cancer, thus supporting the impressive results that PD-1/PD-L1 blockade has led in several cancers (36).

In addition to CTLA-4 and PD-1, other proteins with inhibitory properties, as Lymphocyte-activation gene (LAG)-3, T cell immunoglobulin (TIM)-3, T-cell immunoreceptor with Ig and ITIM domains (TIGIT) are currently under active investigations as potential targets for mAbs (37). LAG-3 is expressed on activated conventional T cells, Tregs, B cells and plasmacytoid dendritic cells (pDCs) (38) and the interaction with its major ligand, Class II MHC, inhibits conventional T cell activity while enhancing the suppressive function of Tregs (39). LAG-3 blockade in addition to anti-PD-1 strategy showed an additive therapeutic activity in preclinical models of chronic infection and cancer, according to their role as markers of exhaustion (40, 41). TIM-3 is another exhaustion-associated inhibitory receptor that blunts T-cell-effector function and induce T cell apoptosis (17). Mouse models of colon adenocarcinoma, melanoma, and sarcoma demonstrated anti-tumor activity of TIM-3 blockade especially in combination with PD-L1 blockade (42, 43). Moreover, anti-TIM-3 treatment increases the proliferation and cytokine production of CD8^+^ T cells derived from patients with melanoma (44). Anti-TIM-3 or anti-PD-L1 mAbs in combination with the blockade of TIGIT, a marker of exhausted cytotoxic cells, showed enhance anti-tumor activity in several animal models (45). Recent studies suggest promise for TIGIT blockade in future immunotherapy regimens without adding significant toxicity (19, 46).

In addition to checkpoint blockade, mAbs targeting agonist receptors, such as inducible co-stimulator (ICOS), OX40 and 4-1BB (47), are currently under clinical development, especially in solid tumors like melanoma (37).

The combined strategy to enhance T-cell activity with co-stimulatory mAbs and concurrently restoring T cell cytotoxic functions against cancer cells by blocking inhibitory proteins could be a promising approach (48). Several clinical trials on both solid and hematological malignancies are currently exploring this strategy (49).

## Regulation of tumor immunity in multiple myeloma and monoclonal gammopathies of undetermined significance

Multiple myeloma (MM) is a hematological malignancy characterized by clonal expansion of terminally differentiated B cells (plasma cells) in the bone marrow (BM). It is clinically manifested with osteolytic bone disease, infections, renal insufficiency, and BM failure (50). The cross talk between malignant plasma cells (PCs) and the BM microenvironment, including immune cells, bone cells, endothelial cells, mesenchymal stromal cells (MSCs) and extracellular matrix, plays a pivotal role in the proliferation and survival of tumor cells (51).

Of note, “immunoparesis,” with a reduction in “uninvolved Igs,” is a common feature of MM (52). PC interactions with BM niche cells create a permissive microenvironment that can promote tumor growth and immune escape, through the production of several factors including TGF-β, interleukin (IL)-10, IL-6, and prostaglandin E2, known to have immunosuppressive properties (53). Among immune cells, DCs display an impaired differentiation and maturation in MM patients (54, 55) and their interaction with PCs enhance MM clonogenicity and proliferation through B cell activating factor (BAFF)/a proliferation inducing ligand (APRIL) signals (56, 57). Malignant PCs can in turn prompt DC fusion and trans-differentiation into osteoclasts (OCs) through receptor activator of nuclear factor κB ligand and CD47 pathways (58–60), thus promoting immunosuppression and disease progression. Beside their role in bone remodeling, OCs also show immunosuppressive properties specifically inducing T-cell apoptosis through the up-regulation of immune checkpoint proteins as TIM-3 and the production of IDO and APRIL (61). These factors increase PD-L1 expression in MM cells thus supporting tumor escape from the immune control (61). DCs can also indirectly enhance osteoclastogenesis by promoting the expansion of T helper 17 (Th-17) clones in MM microenvironment (62) and the consequent accumulation of IL-17, known to be a potent pro-osteoclastogenic factor, in MM BM (60). Sponaas AM et al. reported that myeloid DCs also express PD-L1 and correlate with PD-L1^+^ PCs, suggesting that both cell types could contribute to the suppression of the anti-tumor T cell response in MM through PD-1/PD-L1 pathway (63). Furthermore, MM DC differentiation and maturation is inhibited by MSC production of immunosuppressive factors as IDO, IL-6, PTGS2 (64, 65). MSCs also increase PD-L1 expression on MM cells (66) which in turn suppress PD-1^+^ T cell and NK cell activity (67).

Along with PD-1/PD-L1 axis, a role for other inhibitory pathways, such as CD226 (68), and the induction of T-cell senescence (69) has also been implicated in the suppression of tumor immunity which characterized MM (68, 69). Several studies also reported an accumulation of myeloid derived suppressor cells (MDSCs) and Tregs, along with an unbalanced ratio of Th1/Th2 cells and dysfunctional NK cell cytotoxic activity in MM, compared to patients with monoclonal gammopathy of undetermined significance (MGUS) (70–72). Of note, this loss of function in several immune effector cells is associated with progression to clinical MM (73) and is in part due to the increased expression of suppressive factors, such as ligands of the activating receptor NKG2D (i.e., MHC class I chain-related protein A) from MGUS to MM (74).

More than 10 years ago it was demonstrated that the immune system can detect MGUS pre-neoplastic lesions and potentially control tumor growth (75). Indeed, the presence of CD4^+^ and CD8^+^ T cells, functionally active against pre-neoplastic cells and able to recognize a pattern of specific antigens for each patient tumor, was reported in the BM of MGUS patients (73). A further study identified SOX2 embryonal stem cell antigen as a distinct target of immunity in MGUS compared to MM (76). Interestingly, the presence of SOX2-specific T cells and PD-L1 expression on tumor cells and T cells at baseline was then found to be correlated with the risk of progression to MM (77). Of note, T cells against SOX2 were recently found to be implicated in durable response of a MM patient following chimeric-antigen receptor T (CART) cells (78).

Beside these mechanisms, the establishment of a chronic inflammatory status has been described in the evolution of asymptomatic diseases to MM (79), according to the tight correlation between inflammation and cancer development dating back to Virchow's studies in 1863. It is known that BM serum of MM patients is enriched of pro-inflammatory cytokines, such as IL-1, IL-6, IL-12, IL-15, IL-17, IL-18, IL-22, IL-23, TNF-α, and IFN-γ (80). Moreover, a recent study from Botta C et al. interestingly defined an 8-genes signature (IL8, IL10, IL17A, CCL3, CCL5, VEGFA, EBI3, and NOS2) able to identify MGUS/smoldering/symptomatic-MM with 84% accuracy and built a prognostic risk score based on six genes (IFNG, IL2, LTA, CCL2, VEGFA, CCL3), validated in three additional independent datasets (79).

In the context of MM inflammatory status, bioactive lipids, typically increased during inflammation, may also play a crucial role in tumor development (81). In the past decade, obesity has indeed emerged as one of the risk factors for MM (82) and recent studies have shown an enrichment of lysophosphatidylcholine (LPC) species in MM patient serum compared to healthy donors (HDs) along with an expansion of CD1d-restricted type II NKT cell subsets, reactive against these lipids (83). These cells secrete high amounts of the immunosuppressive IL-13, thus supporting their role in the progression of the disease (83). On the other hand, a decline as well as dysfunctional activation of type I NKT cells was also reported in MM patients, suggesting the balance between these two cell subsets as a new important immune-regulatory axis in the evolution of myeloma (83, 84). In support of this evidence, another study described that CD1d is highly expressed in premalignant and early MM and its expression decreases with disease progression (85). Dysregulation of lipid-reactive immune cells and a higher number of type II NKT cells, with enhanced capacity to promote PC differentiation, may be involved in the increased risk of gammopathy in Gaucher Disease, a lipid disorder (86–88). The multiplicity of mechanisms behind MM immunosuppression and enhancement of disease progression thus suggests the need of combinatorial approaches in the treatment of MM.

## Preclinical studies targeting immune checkpoints in MM

The role of PD-1/PD-L1 pathway in mediating immune escape of malignant PCs and the therapeutic efficacy of PD-1/PD-L1 blockade in other hematological malignancies led to an increased interest in the use of anti-PD-1/PD-L1 therapeutic strategies in MM (68). PD-L1 is highly expressed on PCs isolated from patients with MM, but not on normal PCs (66, 89–91). High PD-L1 expression on PCs was associated with disease progression in patients with MGUS and asymptomatic MM (77) and it could play a role in the development of clonal resistance as demonstrated by PD-L1 high levels in relapsed or refractory MM patients (66). Furthermore, PD-L1 upregulation emerged in patients with minimal residual disease, suggesting that residual PD-L1^+^ myeloma cells have an increased ability to survive and escape immunosurveillance (90). Nevertheless, high variability of PD-1/PD-L1 expression on PCs and BM niche cells was highlighted among patients with the same stage of disease (63, 90).

*In vitro* studies showed that MM microenvironment could induce PD-L1 expression on PCs; PD-L1 up-regulation indeed occurs in the presence of stromal cells (66) and PD-L1 blockade inhibits stromal cell-mediated PC growth (67). This effect is IL-6 dependent and mediated by STAT3, MEK1/2, and JAK2 pathways (66).

IFN-γ produced by cytotoxic T lymphocytes (CTLs) and NK cells strongly induces PD-L1 expression through the activation of MEK/ERK pathway (89). In addition, myeloid DCs, pDCs and MDSCs express PD-L1 in MM patients (63), with an increased proportion of PD-L1^+^ MDSCs in MM patients at remission compared to newly diagnosed and relapsed MM (92).

T cells from MM patients also display higher PD-1 expression levels, associated with loss of effector cell function (93) on both circulating T cells and BM CD8^+^ T and NK cells compared to HDs (67). Moreover, a study from Castella et al. (92) showed that PD-1 expression is already present on the anergic BM Vγ9Vδ2 T cell subset from MGUS patients and remained upregulated in MM after clinical remission (92). In contrast, PD-1 expression is reduced in T cells from patients who achieved minimal disease state following high dose chemotherapy (94).

*In vitro* studies further demonstrated that PD-1/PD-L1 blockade directly enhances NK and T cell mediated anti–MM responses (67, 93) and restores the capacity of PD-L1^+^ pDCs to induce cytotoxic activity of T cells and NK cells against MM PCs (95).

The effects of anti-PD-L1 mAb were also tested *in vivo*, on the 5T33 murine MM models, after autologous (syngeneic) stem-cell transplantation plus administration of a cell-based vaccine (96) or after irradiation (97). It was demonstrated that mice with advanced MM expressed higher levels of PD-1 on both CD8^+^ and CD4^+^ T cells compared to non-tumor bearing mice and the percentages of PD-1^+^ T cells correlated with the amount of tumor burden (97). Moreover, PD-1^+^ CD8^+^ T cells isolated from these mice showed a defective production of pro-inflammatory cytokines (IFN-γ and IL-2) after *in vitro* stimulation and expressed increased levels of the exhausted T cell marker TIM-3 (97). PD-1 blockade also prolonged the survival in disseminated myeloma-bearing mice (90, 96, 97) and this effect was abrogated by the depletion of CD4^+^ or CD8^+^ T cells, thus indicating the main role of both T cell subsets behind this strategy (96). Taken together, these studies supported the potential contribution of PD-1/PD-L1 pathway in the immune escape in MM and suggested that its blockade may be an effective therapeutic strategy against this tumor.

However, current evidences indicate that PD-1 blockade as single agent does not induce clinically meaningful anti-myeloma responses (98). In this regard, it was recently reported that the compromised functions of effector cells in MM may be due to senescence rather than PD-1 mediated exhaustion (69, 98). Exhausted T cells overexpress multiple inhibitory molecules, such as PD-1, CTLA-4, CD160, TIM-3 and LAG-3 and lack of IFN-γ expression (99). However, a PD-1^low^ T cell clonal expansion was observed in 75% of myeloma patients, in contrast to the non-clonal PD-1^high^ T cells (69, 98). This expanded population potentially represented tumor-reactive cells with a senescent phenotype. They indeed showed low levels of LAG-3, TIM-3, PD-1, and CTLA-4 and did not express CD27 and CD28, suggesting a late differentiated phenotype. Moreover, this clone expressed the typical senescent markers CD57, CD160 and KLRG-1 and displayed a secretory profile (69). In addition, it was described that the senescent phenotype was telomere independent as demonstrated by the low levels of p38-mitogen-activated protein kinase, p16 and p21 signaling pathways and it could be potentially reversed by other agents, as immunomodulatory drugs (IMiDs) or histone deacetylase inhibitors (69).

## Immunologic effects of IMiDs- releasing the Ikaros checkpoint

The development of the IMiDs, thalidomide (Thal) and its analogs lenalidomide (Len) and pomalidomide (Pom), has led to a paradigm shift in the treatment of MM (100). IMiDs exert their immunological functions through several mechanisms, including proliferation and functional enhancement of NK/NKT cells, induction of T-cell co-stimulation and reduction of Treg activity, increased Th1 cytokine production, such as IL-2 and IFN-γ, anti-MM ADCC improvement and enhanced DC maturation and functions (101–103). The main molecular mechanism was recently elucidated showing that IMiDs bind Cereblon, causing a subsequent degradation of the transcriptional factors, Ikaros (IKZF1) and Aiolos (IKZF3) on both MM cells and T cells (104). Aiolos is a known repressor of the IL-2 gene promoter while Ikaros is also involved in the regulation of transcriptional silencing during Th2 differentiation (104–106).

Beside these effects, *in vitro* studies interestingly showed that Len treatment downregulates PD-1 expression on both T cells (93) and NK cells (67), restoring their cytotoxic activity, and decreases PD-L1 expression on malignant PCs and MDSCs (93). These data suggested that Len could enhance the effect of anti PD-1/PD-L1 blockade as further reported by Görgün G et al. *In vitro* studies (67).

Moreover, evaluation of immune function in MM patients treated with Pom demonstrated a poly-functional T-cell activation, with increased proportion of co-inhibitory receptor BTLA^+^ T cells and TIM-3^+^ NK cells (107), thus giving a rationale for the use of combination with immune checkpoint inhibitors. Analysis of the molecular mechanism of action revealed that Pom induces depletion of IKZF1 on both T and NK cells; however this effect is dependent on drug exposure and IKZF1 levels return back to baseline, prior to new cycle, with intermittent dosing (107). Interestingly, Pom-mediated immune activation correlated with clinical outcome even in heavily pretreated MM patients; although the baseline expression of Ikaros/Aiolos protein in tumor cells was not predictive of outcome (107).

More recently, a study from Bailur et al. (108) reported that Pom also reduces IKZF1 and IKZF3 levels on innate lymphoid cells (ILCs) and enhances their function, as demonstrated by the increased IFN-γ production both *in vitro* and *in vivo* (108). ILCs are a new subset of innate immune cells known to be involved in the regulation of immunity, inflammation and tissue homeostasis (109). The study also reported that ILCs are increased in BM of MGUS patients compared to HDs and their functions are enhanced in MGUS but decline in patients with asymptomatic MM (108). These results thus provided evidence that ILCs are among the earliest cell subsets enriched in the tumor microenvironment during the evolution of monoclonal gammopathies and represent a possible target to prevent disease progression by acting on their IKZF1 expression. In addition, PD-1 seems to be a negative regulator for ILC function (110) thus supporting the potential for synergy between IMiDs and anti-PD-1 mAbs in the treatment of MM.

## Early clinical studies of checkpoint blockade and combinations in MM

The preclinical evidence that PD-1/PD-L1 blockade enhances T cell and NK cell anti-MM cytotoxicity encouraged the use of mAbs against these checkpoints in clinical trials. However, the use of anti PD-1/PD-L1 antibodies as monotherapy has not provided satisfying results. Specifically, a phase Ib clinical trial testing the anti-PD-1 Nivolumab (IgGk, fully human) in monotherapy reported no objective responses in 27 patients with relapsed or refractory MM (RRMM) (111). Similarly, a phase Ib trial of pembrolizumab (IgGk, humanized anti-PD-1) in monotherapy for RRMM (NCT01953692/KEYNOTE-013) described a stable disease in 57% of patients (112). Preliminary results of a phase II trial of pembrolizumab used in monotherapy as consolidation in MM patients (NCT02636010) demonstrated an increased depth of response in only 3 of 14 patients treated. This lack of efficacy could be explained by the low level of infiltrating effector cells that characterize MM, along with a relatively modest mutational burden as compared to solid tumors wherein therapeutic efficacy correlates with the mutational burden (113).

Lack of single agent activity led to studies testing PD-1/PD-L1 blockade as a part of a combined therapeutic strategy, particularly with IMiDs (Table 2). Pembrolizumab in combination with Len and dexamethasone (Dex) was evaluated in a phase I dose-escalation in 40 RRMM patients who experienced disease progression after more than two prior therapies (114). The objective response rate (ORR) in the whole population was 50%, with an ORR of 38% in Len-refractory patients (114). Preliminary results from the phase II clinical trial conducted on 48 RRMM patients, previously treated with a median of three regimens, showed an ORR of 60%, including 8% of stringent complete response/complete response, 19% VGPR, and 33% PR, with a median duration of response of 14.7 months (115, 116). Interestingly, a phase II study of Pembrolizumab following ASCT reported a CR rate of 31% at 6 months, including a 67% rate of BM MRD-negative state (117).

**Table 2:** Selected clinical trials of checkpoint inhibitor-based therapies in Multiple Myeloma.

**Study**	**Phase**	**Clinical trial identifier**	**References**
A study of pembrolizumab (mk-3475) in combination with standard of care treatments in participants with multiple myeloma (MK-3475-023/KEYNOTE-023)	I	NCT02036502	(114)
An investigational immuno-therapy study to determine the safety and effectiveness of nivolumab and daratumumab, with or without pomalidomide and dexamethasone, in patients with multiple myeloma	I	NCT01592370	(118)
Study of lenalidomide/dexamethasone with nivolumab and ipilimumab in patients with newly diagnosed multiple myeloma	I	NCT03283046	–
A study to determine dose and regimen of durvalumab as monotherapy or in combination with pomalidomide with or without dexamethasone in subjects with relapsed and refractory multiple myeloma	I	NCT02616640	–
A study of PVX-410, a cancer vaccine, and durvalumab ± lenalidomide for smoldering MM	I	NCT02886065	–
A study of atezolizumab (anti-programmed death-ligand 1 [PD-L1] antibody) alone or in combination with an immunomodulatory drug and/or daratumumab in participants with multiple myeloma (MM)	Ib	NCT02431208	–
A study of durvalumab in combination with lenalidomide with and without dexamethasone in subjects with newly diagnosed multiple myeloma	Ib	NCT02685826	–
1454GCC: Anti-PD-1 (MK-3475) and IMiD (Pomalidomide) combination immunotherapy in relapsed/refractory multiple myeloma	I/II	NCT02289222	(115, 116)
Pembrolizumab cyclophosphamide and lenalidomide for patients with relapsed multiple myeloma (MUKfourteen)	I/II	NCT03191981	–
Pembrolizumab, lenalidomide, and dexamethasone in treating patients with newly diagnosed multiple myeloma eligible for stem cell transplant	II	NCT02880228	–
Phase 2 multi-center study of anti-pd-1 during lymphopenic state after HDT/ASCT for multiple myeloma	II	NCT02331368	(117)
Pembrolizumab + Lenalidomide post-autologous stem cell transplant (ASCT) in high-risk multiple myeloma (MM)	II	NCT02906332	(119)
Efficacy and safety study of pembrolizumab (MK-3475) in combination with daratumumab in participants with relapsed refractory multiple myeloma (MK-3475-668/KEYNOTE-668)	II	KEYNOTE-668 NCT03221634	–
A study of elotuzumab in combination with pomalidomide and low dose dexamethasone and elotuzumab in combination with nivolumab in patients with multiple myeloma relapsed or refractory to prior treatment with lenalidomide	II	NCT02612779	–
An exploratory study to evaluate the combination of elotuzumab and nivolumab with and without pomalidomide in relapsed refractory multiple myeloma	II	NCT03227432	–
A Phase II trial if nivolumab, lenalidomide and dexamethasone in high risk smoldering myeloma	II	NCT02903381	Based on ClinicalTrials.gov. U.S. National Library of Medicine [https://clinicaltrials.gov/]. Accessed 2 Jan 2018.
A study to determine the safety and efficacy for the combination of durvalumab and daratumumab in relapsed and refractory multiple myeloma (FUSIONMM-003)	II	NCT02807454	–
A study to determine the efficacy of the combination of Daratumumab (DARA) plus Durvalumab (DURVA) (D2) in subjects with Relapsed and Refractory Multiple Myeloma (RRMM) (FUSION-MM-005)	II	NCT03000452	–
Study of pomalidomide and low dose dexamethasone with or without pembrolizumab (MK-3475) in Refractory or Relapsed and Refractory Multiple Myeloma (rrMM) (MK3475-183/KEYNOTE-183)	III	KEYNOTE-183/NCT02576977	https://www.fda.gov/Drugs/DrugSafety/ucm574305.htm
Study of lenalidomide and dexamethasone with or without pembrolizumab (MK-3475) in participants with newly diagnosed treatment naive multiple myeloma (MK3475-185/KEYNOTE-185)	III	KEYNOTE-185/NCT02579863	https://www.fda.gov/Drugs/DrugSafety/ucm574305.htm

These results lead to the development of the phase III studies of pembrolizumab in combination with Len and Dex (KEYNOTE-185, NCT02579863) or Pom and Dex (KEYNOTE-183, NCT02576977) and one phase III study of Pom and Dex vs. nivolumab, Pom, and Dex vs. nivolumab, elotuzumab, Pom, and Dex (CheckMate 602, NCT02726581). However, in June 2017 the US Food and Drug Administration transiently halted the clinical trials of anti-PD-1/PD-L1 mAbs in combination with IMiDs, due to an imbalance of deaths in the Pembrolizumab arms in KEYNOTE-183 and KEYNOTE-185 and no significant differences in terms of objective response (https://www.fda.gov/Drugs/DrugSafety/ucm574305.htm). As these studies have not yet been published in a peer-reviewed format, more details that might shed light on the possible explanations for these observations are lacking. With further review of safety data on ongoing trials, some of the studies of combinations of PD-1/PD-L1 blockade in MM have now been reinitiated. Combinations of PD-1 blockade with other MM therapies are also currently under evaluation. Preliminary results on a phase I trial of the anti PD-1 Nivolumab in combination with other established anti-myeloma agents (Len/Pom, Dex, anti-CD38 Daratumumab, proteasome inhibitors) revealed acceptable safety profile in refractory, heavily pre-treated, high-risk MM patients (118). In addition anti-PD-1 based therapy, clinical trials of mAbs targeting PD-L1 (Atezolizumab and Durvalumab), both alone and in combination with other agents (i.e., Elotuzumab, and Daratumumab) have also been developed.

Together, these studies point to the need for careful evaluation of immune checkpoint strategies and their combinations in MM, with cautious attention to toxicities as well as pharmacodynamics endpoints.

## Major unmet needs and future directions

The concept that immune system can regulate the growth of MM cells is now well-established and immune-based approaches carry the promise of long term disease control and even cure without the need for ongoing therapy. Current MM therapies, such as IMiDs and anti-CD38 antibodies can have immunologic effects; newer therapies particularly CAR-T cells and T cell-engaging bi-specifics are in active clinical investigation and showing promising results. However, there remains an unmet need to address the mechanisms operative in the tumor microenvironment that restrict or prevent long term control of tumors.

Further studies are needed to better understand the mechanisms behind the lack of clinical activity of single agent PD-1 blockade in MM. Several mechanistic possibilities exist, including dominance of other inhibitory checkpoints, immune suppressive cells, lack of agonistic signaling, the low number of tumor-specific T cells in the tumor microenvironment, poor antigen presentation, low mutational burden of MM tumors, as well as senescence of tumor-infiltrating T cells. Moreover, MM is not a single disease and it consists of several distinct genetic subtypes; thus, it is likely that immune microenvironment in MM may also differ between patients. This heterogeneity may even be spatial within the same patient, as recently illustrated for solid tumors (120). As MM is a malignancy involving an immune cell, it is also theoretically possible that PD-1 blockade may lead to altered cross-talk with other immune cells and paradoxically promote tumor growth. It is also of interest to identify if there are specific subsets of patients (such as those with high mutational burden on MM cells), who might preferentially benefit from checkpoint blockade.

Some of the possibilities discussed above suggest the chance that the lack of efficacy of PD-1 blocking antibodies as single agents can be reverted by the combination with other agents. This strategy could lead to distinct pharmacodynamics effects as well as toxicity profiles compared to monotherapies (121).

It should be noted that many of the published data involve PD-1 targeted therapies; however, the effects of PD-L1 blockade may differ.

Preclinical studies also suggest a potential efficacy of agonistic antibodies in preclinical models as well. As an example, anti-CD137 Abs were shown to lead to strong tumor immunity in VKappa-myc MM models (122, 123). Although a small study with this agent in MM was not completed, further evaluation of this pathway particularly in combination may be of interest. T cells in MM lesions also express other inhibitory molecules, such as TIM-3 and LAG-3. Antibodies targeting these molecules are now entering the clinic and the effects of these agents in human MM are awaited. In addition to their effects on T cells, immune regulatory pathways are also operative for innate cells, such as NK-T cells and ILCs. These pathways may also limit the efficacy of engineered T cells, such as CAR-T cells, as well as bispecifics. Future combinations of these strategies to harness immune-mediated MM control are therefore eagerly awaited.

## Author contributions

FC wrote the first draft of the paper; FC, RD, JKB, KD, and MVD reviewed the paper and gave final approval of the submitted publication.

### Conflict of interest statement

MVD has served on advisory boards for BMS, Merck and Genentech/Roche. The remaining authors declare that the research was conducted in the absence of any commercial or financial relationships that could be construed as a potential conflict of interest.

## References

[1] Coley WB. The treatment of inoperable sarcoma by bacterial toxins (the Mixed Toxins of the Streptococcus erysipelas and the Bacillus prodigiosus). Proc R Soc Med. (1910) 3:1–48. 19974799 10.1177/003591571000301601 PMC1961042

[2] Khalil DN Smith EL Brentjens RJ Wolchok JD The future of cancer treatment: immunomodulation, CARs and combination immunotherapy. Nat Rev Clin Oncol. (2016) 13:273–90. 10.1038/nrclinonc.2016.25 26977780 PMC5551685

[3] Dunn GP Bruce AT Ikeda H Old LJ Schreiber RD. Cancer immunoediting: from immunosurveillance to tumor escape. Nat Immunol. (2002) 3:991–8. 10.1038/ni1102-991 12407406

[4] Hanahan D Weinberg RA. The hallmarks of cancer. Cell (2000) 100:57–70. 10.1016/S0092-8674(00)81683-9 10647931

[5] Maio M Covre A Fratta E Di Giacomo AM Taverna P Natali PG. Molecular pathways: at the crossroads of cancer epigenetics and immunotherapy. Clin Cancer Res. (2015) 21:4040–7. 10.1158/1078-0432.CCR-14-2914 26374074

[6] Peng D Kryczek I Nagarsheth N Zhao L Wei S Wang W. Epigenetic silencing of TH1-type chemokines shapes tumour immunity and immunotherapy. Nature (2015) 527:249–53. 10.1038/nature15520 26503055 PMC4779053

[7] Schreiber RD Old LJ Smyth MJ. Cancer immunoediting: integrating immunity's roles in cancer suppression and promotion. Science (2011) 331:1565–70. 10.1126/science.1203486 21436444

[8] Vesely MD Kershaw MH Schreiber RD Smyth MJ. Natural innate and adaptive immunity to cancer. Annu Rev Immunol. (2011) 29:235–71. 10.1146/annurev-immunol-031210-101324 21219185

[9] Dunn GP Koebel CM Schreiber RD. Interferons, immunity and cancer immunoediting. Nat Rev Immunol. (2006) 6:836–48. 10.1038/nri1961 17063185

[10] Finn OJ. A believer's overview of cancer immunosurveillance and immunotherapy. J Immunol. (2018) 200:385–91. 10.4049/jimmunol.1701302 29311379 PMC5763509

[11] Dyck L Mills KHG. Immune checkpoints and their inhibition in cancer and infectious diseases. Eur J Immunol. (2017) 47:765–79. 10.1002/eji.201646875 28393361

[12] Engelhardt JJ Sullivan TJ Allison JP. CTLA-4 overexpression inhibits T cell responses through a CD28-B7-dependent mechanism. J Immunol. (2006) 177:1052–61. 10.4049/jimmunol.177.2.1052 16818761

[13] Peggs KS Quezada SA Chambers CA Korman AJ Allison JP. Blockade of CTLA-4 on both effector and regulatory T cell compartments contributes to the antitumor activity of anti-CTLA-4 antibodies. J Exp Med. (2009) 206:1717–25. 10.1084/jem.20082492 19581407 PMC2722174

[14] Keir ME Butte MJ Freeman GJ Sharpe AH. PD-1 and its ligands in tolerance and immunity. Annu Rev Immunol. (2008) 26:677–704. 10.1146/annurev.immunol.26.021607.090331 18173375 PMC10637733

[15] Huang CT Workman CJ Flies D Pan X Marson AL Zhou G. Role of LAG-3 in regulatory T cells. Immunity (2004) 21:503–13. 10.1016/j.immuni.2004.08.010 15485628

[16] Zhu C Anderson AC Schubart A Xiong H Imitola J Khoury SJ. The Tim-3 ligand galectin-9 negatively regulates T helper type 1 immunity. Nat Immunol. (2005) 6:1245–52. 10.1038/ni1271 16286920

[17] Jin HT Anderson AC Tan WG West EE Ha SJ Araki K. Cooperation of Tim-3 and PD-1 in CD8 T-cell exhaustion during chronic viral infection. Proc Natl Acad Sci USA. (2010) 107:14733–8. 10.1073/pnas.1009731107 20679213 PMC2930455

[18] Yu X Harden K Gonzalez LC Francesco M Chiang E Irving B. The surface protein TIGIT suppresses T cell activation by promoting the generation of mature immunoregulatory dendritic cells. Nat Immunol. (2009) 10:48–57. 10.1038/ni.1674 19011627

[19] Joller N Hafler JP Brynedal B Kassam N Spoerl S Levin SD. Cutting edge: TIGIT has T cell-intrinsic inhibitory functions. J Immunol. (2011) 186:1338–42. 10.4049/jimmunol.1003081 21199897 PMC3128994

[20] Pardoll DM. The blockade of immune checkpoints in cancer immunotherapy. Nat Rev Cancer (2012) 12:252–64. 10.1038/nrc3239 22437870 PMC4856023

[21] Pico De Coana Y Choudhury A Kiessling R Checkpoint blockade for cancer therapy: revitalizing a suppressed immune system. Trends Mol Med. (2015) 21:482–91. 10.1016/j.molmed.2015.05.005 26091825

[22] Robert C Long GV Brady B Dutriaux C Maio M Mortier L. Nivolumab in previously untreated melanoma without BRAF mutation. N Engl J Med. (2015) 372:320–30. 10.1056/NEJMoa1412082 25399552

[23] Robert C Schachter J Long GV Arance A Grob JJ Mortier L. Pembrolizumab versus ipilimumab in advanced melanoma. N Engl J Med. (2015) 372:2521–32. 10.1056/NEJMoa1503093 25891173

[24] Wolchok JD Saenger Y. The mechanism of anti-CTLA-4 activity and the negative regulation of T-cell activation. Oncologist (2008) 13(Suppl 4):2–9. 10.1634/theoncologist.13-S4-2 19001145

[25] Alegre ML Frauwirth KA Thompson CB. T-cell regulation by CD28 and CTLA-4. Nat Rev Immunol. (2001) 1:220–8. 10.1038/35105024 11905831

[26] Linsley PS Greene JL Brady W Bajorath J Ledbetter JA Peach R. Human B7-1 (CD80) and B7-2 (CD86) bind with similar avidities but distinct kinetics to CD28 and CTLA-4 receptors. Immunity (1994) 1:793–801. 10.1016/S1074-7613(94)80021-9 7534620

[27] Rudd CE Taylor A Schneider H. CD28 and CTLA-4 coreceptor expression and signal transduction. Immunol Rev. (2009) 229:12–26. 10.1111/j.1600-065X.2009.00770.x 19426212 PMC4186963

[28] Halpert MM Konduri V Liang D Chen Y Wing JB Paust S. Dendritic cell-secreted cytotoxic T-lymphocyte-associated protein-4 regulates the T-cell response by downmodulating bystander surface B7. Stem Cells Dev. (2016) 25:774–87. 10.1089/scd.2016.0009 26979751 PMC4870609

[29] Topalian SL Drake CG Pardoll DM. Immune checkpoint blockade: a common denominator approach to cancer therapy. Cancer Cell (2015) 27:450–61. 10.1016/j.ccell.2015.03.001 25858804 PMC4400238

[30] Chemnitz JM Parry RV Nichols KE June CH Riley JL. SHP-1 and SHP-2 associate with immunoreceptor tyrosine-based switch motif of programmed death 1 upon primary human T cell stimulation, but only receptor ligation prevents T cell activation. J Immunol. (2004) 173:945–54. 10.4049/jimmunol.173.2.945 15240681

[31] Parry RV Chemnitz JM Frauwirth KA Lanfranco AR Braunstein I Kobayashi SV. CTLA-4 and PD-1 receptors inhibit T-cell activation by distinct mechanisms. Mol Cell Biol. (2005) 25:9543–53. 10.1128/MCB.25.21.9543-9553.2005 16227604 PMC1265804

[32] Freeman GJ Long AJ Iwai Y Bourque K Chernova T Nishimura H. Engagement of the PD-1 immunoinhibitory receptor by a novel B7 family member leads to negative regulation of lymphocyte activation. J Exp Med. (2000) 192:1027–34. 10.1084/jem.192.7.1027 11015443 PMC2193311

[33] Dong H Zhu G Tamada K Flies DB Van Deursen JM Chen L. B7-H1 determines accumulation and deletion of intrahepatic CD8(+) T lymphocytes. Immunity (2004) 20:327–36. 10.1016/S1074-7613(04)00050-0 15030776

[34] Francisco LM Sage PT Sharpe AH. The PD-1 pathway in tolerance and autoimmunity. Immunol Rev. (2010) 236:219–42. 10.1111/j.1600-065X.2010.00923.x 20636820 PMC2919275

[35] Parsa AT Waldron JS Panner A Crane CA Parney IF Barry JJ. Loss of tumor suppressor PTEN function increases B7-H1 expression and immunoresistance in glioma. Nat Med. (2007) 13:84–8. 10.1038/nm1517 17159987

[36] Okazaki T Honjo T. PD-1 and PD-1 ligands: from discovery to clinical application. Int Immunol. (2007) 19:813–24. 10.1093/intimm/dxm057 17606980

[37] Sharma P Allison JP. The future of immune checkpoint therapy. Science (2015) 348:56–61. 10.1126/science.aaa8172 25838373

[38] Huard B Gaulard P Faure F Hercend T Triebel F. Cellular expression and tissue distribution of the human LAG-3-encoded protein, an MHC class II ligand. Immunogenetics (1994) 39:213–7. 10.1007/BF00241263 7506235

[39] Huard B Prigent P Tournier M Bruniquel D Triebel F. CD4/major histocompatibility complex class II interaction analyzed with CD4- and lymphocyte activation gene-3 (LAG-3)-Ig fusion proteins. Eur J Immunol. (1995) 25:2718–21. 10.1002/eji.1830250949 7589152

[40] Blackburn SD Shin H Haining WN Zou T Workman CJ Polley A. Coregulation of CD8^+^ T cell exhaustion by multiple inhibitory receptors during chronic viral infection. Nat Immunol. (2009) 10:29–37. 10.1038/ni.1679 19043418 PMC2605166

[41] Woo SR Turnis ME Goldberg MV Bankoti J Selby M Nirschl CJ. Immune inhibitory molecules LAG-3 and PD-1 synergistically regulate T-cell function to promote tumoral immune escape. Cancer Res. (2012) 72:917–27. 10.1158/0008-5472.CAN-11-1620 22186141 PMC3288154

[42] Sakuishi K Apetoh L Sullivan JM Blazar BR Kuchroo VK Anderson AC. Targeting Tim-3 and PD-1 pathways to reverse T cell exhaustion and restore anti-tumor immunity. J Exp Med. (2010) 207:2187–94. 10.1084/jem.20100643 20819927 PMC2947065

[43] Ngiow SF Von Scheidt B Akiba H Yagita H Teng MW Smyth MJ. Anti-TIM3 antibody promotes T cell IFN-gamma-mediated antitumor immunity and suppresses established tumors. Cancer Res. (2011) 71:3540–51. 10.1158/0008-5472.CAN-11-0096 21430066

[44] Fourcade J Sun Z Benallaoua M Guillaume P Luescher IF Sander C. Upregulation of Tim-3 and PD-1 expression is associated with tumor antigen-specific CD8^+^ T cell dysfunction in melanoma patients. J Exp Med. (2010) 207:2175–86. 10.1084/jem.20100637 20819923 PMC2947081

[45] Anderson AC Joller N Kuchroo VK. Lag-3, Tim-3, and TIGIT: co-inhibitory receptors with specialized functions in immune regulation. Immunity (2016) 44:989–1004. 10.1016/j.immuni.2016.05.001 27192565 PMC4942846

[46] Dixon KO Schorer M Nevin J Etminan Y Amoozgar Z Kondo T. Functional anti-TIGIT antibodies regulate development of autoimmunity and antitumor immunity. J Immunol. (2018) 200:3000–7. 10.4049/jimmunol.1700407 29500245 PMC5893394

[47] Buchan SL Rogel A Al-Shamkhani A. The immunobiology of CD27 and OX40 and their potential as targets for cancer immunotherapy. Blood (2018) 131:39–48. 10.1182/blood-2017-07-741025 29118006

[48] Buchan SL Fallatah M Thirdborough SM Taraban VY Rogel A Thomas LJ. PD-1 blockade and CD27 stimulation activate distinct transcriptional programs that synergize for CD8(+) T-cell-driven antitumor immunity. Clin Cancer Res. (2018) 24:2383–94. 10.1158/1078-0432.CCR-17-3057 29514845 PMC5959006

[49] Naidoo J Page DB Wolchok JD. Immune modulation for cancer therapy. Br J Cancer (2014) 111:2214–9. 10.1038/bjc.2014.348 25211661 PMC4264429

[50] Kyle RA Rajkumar SV. Multiple myeloma. N Engl J Med. (2004) 351:1860–73. 10.1056/NEJMra041875 15509819

[51] Anderson KC Carrasco RD. Pathogenesis of myeloma. Annu Rev Pathol. (2011) 6:249–74. 10.1146/annurev-pathol-011110-130249 21261519

[52] Perez-Persona E Vidriales MB Mateo G Garcia-Sanz R Mateos MV De Coca AG. New criteria to identify risk of progression in monoclonal gammopathy of uncertain significance and smoldering multiple myeloma based on multiparameter flow cytometry analysis of bone marrow plasma cells. Blood (2007) 110:2586–92. 10.1182/blood-2007-05-088443 17576818

[53] Pratt G Goodyear O Moss P. Immunodeficiency and immunotherapy in multiple myeloma. Br J Haematol. (2007) 138:563–79. 10.1111/j.1365-2141.2007.06705.x 17686051

[54] Ratta M Fagnoni F Curti A Vescovini R Sansoni P Oliviero B. Dendritic cells are functionally defective in multiple myeloma: the role of interleukin-6. Blood (2002) 100:230–7. 10.1182/blood.V100.1.230 12070032

[55] Bahlis NJ King AM Kolonias D Carlson LM Liu HY Hussein MA. CD28-mediated regulation of multiple myeloma cell proliferation and survival. Blood (2007) 109:5002–10. 10.1182/blood-2006-03-012542 17311991 PMC1885531

[56] Kukreja A Hutchinson A Dhodapkar K Mazumder A Vesole D Angitapalli R. Enhancement of clonogenicity of human multiple myeloma by dendritic cells. J Exp Med. (2006) 203:1859–65. 10.1084/jem.20052136 16880256 PMC2034506

[57] Chauhan D Singh AV Brahmandam M Carrasco R Bandi M Hideshima T. Functional interaction of plasmacytoid dendritic cells with multiple myeloma cells: a therapeutic target. Cancer Cell (2009) 16:309–23. 10.1016/j.ccr.2009.08.019 19800576 PMC2762396

[58] Kukreja A Radfar S Sun BH Insogna K Dhodapkar MV. Dominant role of CD47-thrombospondin-1 interactions in myeloma-induced fusion of human dendritic cells: implications for bone disease. Blood (2009) 114:3413–21. 10.1182/blood-2009-03-211920 19661269 PMC2765677

[59] Tucci M Ciavarella S Strippoli S Brunetti O Dammacco F Silvestris F. Immature dendritic cells from patients with multiple myeloma are prone to osteoclast differentiation *in vitro*. Exp Hematol. (2011) 39:773–83.e1. 10.1016/j.exphem.2011.04.006 21569821

[60] Tucci M Stucci S Strippoli S Dammacco F Silvestris F Dendritic cells and malignant plasma cells: an alliance in multiple myeloma tumor progression? Oncologist (2011) 16:1040–8. 10.1634/theoncologist.2010-0327 21659611 PMC3228131

[61] An G Acharya C Feng X Wen K Zhong M Zhang L. Osteoclasts promote immune suppressive microenvironment in multiple myeloma: therapeutic implication. Blood (2016) 128:1590–603. 10.1182/blood-2016-03-707547 27418644 PMC5034739

[62] Dhodapkar KM Barbuto S Matthews P Kukreja A Mazumder A Vesole D. Dendritic cells mediate the induction of polyfunctional human IL17-producing cells (Th17-1 cells) enriched in the bone marrow of patients with myeloma. Blood (2008) 112:2878–85. 10.1182/blood-2008-03-143222 18669891 PMC2556623

[63] Sponaas AM Moharrami NN Feyzi E Standal T Holth Rustad E Waage A. PDL1 expression on plasma and dendritic cells in myeloma bone marrow suggests benefit of targeted anti PD1-PDL1 therapy. PLoS ONE (2015) 10:e0139867. 10.1371/journal.pone.0139867 26444869 PMC4596870

[64] Nauta AJ Fibbe WE. Immunomodulatory properties of mesenchymal stromal cells. Blood (2007) 110:3499–506. 10.1182/blood-2007-02-069716 17664353

[65] Spaggiari GM Abdelrazik H Becchetti F Moretta L. MSCs inhibit monocyte-derived DC maturation and function by selectively interfering with the generation of immature DCs: central role of MSC-derived prostaglandin E2. Blood (2009) 113:6576–83. 10.1182/blood-2009-02-203943 19398717

[66] Tamura H Ishibashi M Yamashita T Tanosaki S Okuyama N Kondo A. Marrow stromal cells induce B7-H1 expression on myeloma cells, generating aggressive characteristics in multiple myeloma. Leukemia (2013) 27:464–72. 10.1038/leu.2012.213 22828443

[67] Gorgun G Samur MK Cowens KB Paula S Bianchi G Anderson JE. Lenalidomide enhances immune checkpoint blockade-induced immune response in multiple myeloma. Clin Cancer Res. (2015) 21:4607–18. 10.1158/1078-0432.CCR-15-0200 25979485 PMC4609232

[68] Guillerey C Ferrari De Andrade L Vuckovic S Miles K Ngiow SF Yong MC Immunosurveillance and therapy of multiple myeloma are CD226 dependent. J Clin Invest. (2015) 125:2077–89. 10.1172/JCI77181 25893601 PMC4463191

[69] Suen H Brown R Yang S Weatherburn C Ho PJ Woodland N. Multiple myeloma causes clonal T-cell immunosenescence: identification of potential novel targets for promoting tumour immunity and implications for checkpoint blockade. Leukemia (2016) 30:1716–24. 10.1038/leu.2016.84 27102208

[70] Brimnes MK Vangsted AJ Knudsen LM Gimsing P Gang AO Johnsen HE. Increased level of both CD4^+^FOXP3^+^ regulatory T cells and CD14^+^HLA-DR(-)/low myeloid-derived suppressor cells and decreased level of dendritic cells in patients with multiple myeloma. Scand J Immunol. (2010) 72:540–7. 10.1111/j.1365-3083.2010.02463.x 21044128

[71] Gorgun GT Whitehill G Anderson JL Hideshima T Maguire C Laubach J. Tumor-promoting immune-suppressive myeloid-derived suppressor cells in the multiple myeloma microenvironment in humans. Blood (2013) 121:2975–87. 10.1182/blood-2012-08-448548 23321256 PMC3624943

[72] Romano A Conticello C Cavalli M Vetro C La Fauci A Parrinello NL. Immunological dysregulation in multiple myeloma microenvironment. Biomed Res Int. (2014) 2014:198539. 10.1155/2014/198539 25013764 PMC4071780

[73] Dhodapkar MV Geller MD Chang DH Shimizu K Fujii S Dhodapkar KM. A reversible defect in natural killer T cell function characterizes the progression of premalignant to malignant multiple myeloma. J Exp Med. (2003) 197:1667–76. 10.1084/jem.20021650 12796469 PMC2193955

[74] Jinushi M Vanneman M Munshi NC Tai YT Prabhala RH Ritz J. MHC class I chain-related protein A antibodies and shedding are associated with the progression of multiple myeloma. Proc Natl Acad Sci USA. (2008) 105:1285–90. 10.1073/pnas.0711293105 18202175 PMC2234130

[75] Dhodapkar MV. Harnessing host immune responses to preneoplasia: promise and challenges. Cancer Immunol Immunother. (2005) 54:409–13. 10.1007/s00262-004-0607-8 15602654 PMC11033007

[76] Spisek R Charalambous A Mazumder A Vesole DH Jagannath S Dhodapkar MV. Bortezomib enhances dendritic cell (DC)-mediated induction of immunity to human myeloma via exposure of cell surface heat shock protein 90 on dying tumor cells: therapeutic implications. Blood (2007) 109:4839–45. 10.1182/blood-2006-10-054221 17299090 PMC1885516

[77] Dhodapkar MV Sexton R Das R Dhodapkar KM Zhang L Sundaram R. Prospective analysis of antigen-specific immunity, stem-cell antigens, and immune checkpoints in monoclonal gammopathy. Blood (2015) 126:2475–8. 10.1182/blood-2015-03-632919 26468228 PMC4661169

[78] Garfall AL Stadtmauer EA Hwang WT Lacey SF Melenhorst JJ Krevvata M. Anti-CD19 CAR T cells with high-dose melphalan and autologous stem cell transplantation for refractory multiple myeloma. JCI Insight. (2018) 3:120505. 10.1172/jci.insight.120505 29669947 PMC5931130

[79] Botta C Di Martino MT Ciliberto D Cuce M Correale P Rossi M. A gene expression inflammatory signature specifically predicts multiple myeloma evolution and patients survival. Blood Cancer J. (2016) 6:e511. 10.1038/bcj.2016.118 27983725 PMC5223153

[80] Musolino C Allegra A Innao V Allegra AG Pioggia G Gangemi S. Inflammatory and anti-inflammatory equilibrium, proliferative and antiproliferative balance: the role of cytokines in multiple myeloma. Mediators Inflamm. (2017) 2017:1852517. 10.1155/2017/1852517 29089667 PMC5635476

[81] Dhodapkar MV Richter J. Harnessing natural killer T (NKT) cells in human myeloma: progress and challenges. Clin Immunol. (2011) 140:160–6. 10.1016/j.clim.2010.12.010 21233022 PMC3224820

[82] Larsson SC Wolk A. Body mass index and risk of multiple myeloma: a meta-analysis. Int J Cancer (2007) 121:2512–6. 10.1002/ijc.22968 17680557

[83] Chang DH Deng H Matthews P Krasovsky J Ragupathi G Spisek R. Inflammation-associated lysophospholipids as ligands for CD1d-restricted T cells in human cancer. Blood (2008) 112:1308–16. 10.1182/blood-2008-04-149831 18535199 PMC2515141

[84] Richter J Neparidze N Zhang L Nair S Monesmith T Sundaram R. Clinical regressions and broad immune activation following combination therapy targeting human NKT cells in myeloma. Blood (2013) 121:423–30. 10.1182/blood-2012-06-435503 23100308 PMC3548165

[85] Spanoudakis E Hu M Naresh K Terpos E Melo V Reid A. Regulation of multiple myeloma survival and progression by CD1d. Blood (2009) 113:2498–507. 10.1182/blood-2008-06-161281 19056691

[86] Nair S Boddupalli CS Verma R Liu J Yang R Pastores GM. Type II NKT-TFH cells against Gaucher lipids regulate B-cell immunity and inflammation. Blood (2015) 125:1256–71. 10.1182/blood-2014-09-600270 25499455 PMC4335081

[87] Nair S Branagan AR Liu J Boddupalli CS Mistry PK Dhodapkar MV. Clonal immunoglobulin against lysolipids in the origin of myeloma. N Engl J Med. (2016) 374:555–61. 10.1056/NEJMoa1508808 26863356 PMC4804194

[88] Nair S Sng J Boddupalli CS Seckinger A Chesi M Fulciniti M. Antigen-mediated regulation in monoclonal gammopathies and myeloma. JCI Insight (2018) 3:e98259. 10.1172/jci.insight.98259 29669929 PMC5931125

[89] Liu J Hamrouni A Wolowiec D Coiteux V Kuliczkowski K Hetuin D. Plasma cells from multiple myeloma patients express B7-H1 (PD-L1) and increase expression after stimulation with IFN-{gamma} and TLR ligands via a MyD88-, TRAF6-, and MEK-dependent pathway. Blood (2007) 110:296–304. 10.1182/blood-2006-10-051482 17363736

[90] Paiva B Azpilikueta A Puig N Ocio EM Sharma R Oyajobi BO. PD-L1/PD-1 presence in the tumor microenvironment and activity of PD-1 blockade in multiple myeloma. Leukemia (2015) 29:2110–3. 10.1038/leu.2015.79 25778100

[91] Yousef S Marvin J Steinbach M Langemo A Kovacsovics T Binder M. Immunomodulatory molecule PD-L1 is expressed on malignant plasma cells and myeloma-propagating pre-plasma cells in the bone marrow of multiple myeloma patients. Blood Cancer J. (2015) 5:e285. 10.1038/bcj.2015.7 25747678 PMC4382668

[92] Castella B Foglietta M Sciancalepore P Rigoni M Coscia M Griggio V. Anergic bone marrow Vgamma9Vdelta2 T cells as early and long-lasting markers of PD-1-targetable microenvironment-induced immune suppression in human myeloma. Oncoimmunology (2015) 4:e1047580. 10.1080/2162402X.2015.1047580 26451323 PMC4589055

[93] Benson DMJr Bakan CE Mishra A Hofmeister CC Efebera Y Becknell B. The PD-1/PD-L1 axis modulates the natural killer cell versus multiple myeloma effect: a therapeutic target for CT-011, a novel monoclonal anti-PD-1 antibody. Blood (2010) 116:2286–94. 10.1182/blood-2010-02-271874 20460501 PMC3490105

[94] Rosenblatt J Glotzbecker B Mills H Vasir B Tzachanis D Levine JD. PD-1 blockade by CT-011, anti-PD-1 antibody, enhances *ex vivo* T-cell responses to autologous dendritic cell/myeloma fusion vaccine. J Immunother. (2011) 34:409–18. 10.1097/CJI.0b013e31821ca6ce 21577144 PMC3142955

[95] Ray A Das DS Song Y Richardson P Munshi NC Chauhan D. Targeting PD1-PDL1 immune checkpoint in plasmacytoid dendritic cell interactions with T cells, natural killer cells and multiple myeloma cells. Leukemia (2015) 29:1441–4. 10.1038/leu.2015.11 25634684 PMC5703039

[96] Kearl TJ Jing W Gershan JA Johnson BD. Programmed death receptor-1/programmed death receptor ligand-1 blockade after transient lymphodepletion to treat myeloma. J Immunol. (2013) 190:5620–8. 10.4049/jimmunol.1202005 23616570 PMC3891840

[97] Hallett WH Jing W Drobyski WR Johnson BD. Immunosuppressive effects of multiple myeloma are overcome by PD-L1 blockade. Biol Blood Marrow Transplant. (2011) 17:1133–45. 10.1016/j.bbmt.2011.03.011 21536144

[98] Suen H Brown R Yang S Ho PJ Gibson J Joshua D. The failure of immune checkpoint blockade in multiple myeloma with PD-1 inhibitors in a phase 1 study. Leukemia (2015) 29:1621–2. 10.1038/leu.2015.104 25987102

[99] Crespo J Sun H Welling TH Tian Z Zou W. T cell anergy, exhaustion, senescence, and stemness in the tumor microenvironment. Curr Opin Immunol. (2013) 25:214–21. 10.1016/j.coi.2012.12.003 23298609 PMC3636159

[100] Palumbo A Anderson K. Multiple myeloma. N Engl J Med. (2011) 364:1046–60. 10.1056/NEJMra1011442 21410373

[101] Galustian C Meyer B Labarthe MC Dredge K Klaschka D Henry J. The anti-cancer agents lenalidomide and pomalidomide inhibit the proliferation and function of T regulatory cells. Cancer Immunol Immunother. (2009) 58:1033–45. 10.1007/s00262-008-0620-4 19009291 PMC11030759

[102] Luptakova K Rosenblatt J Glotzbecker B Mills H Stroopinsky D Kufe T. Lenalidomide enhances anti-myeloma cellular immunity. Cancer Immunol Immunother. (2013) 62:39–49. 10.1007/s00262-012-1308-3 22733396 PMC4098790

[103] Costa F Vescovini R Bolzoni M Marchica V Storti P Toscani D. Lenalidomide increases human dendritic cell maturation in multiple myeloma patients targeting monocyte differentiation and modulating mesenchymal stromal cell inhibitory properties. Oncotarget (2017) 8:53053–67. 10.18632/oncotarget.18085 28881793 PMC5581092

[104] Kronke J Hurst SN Ebert BL. Lenalidomide induces degradation of IKZF1 and IKZF3. Oncoimmunology (2014) 3:e941742. 10.4161/21624011.2014.941742 25610725 PMC4292522

[105] Thomas RM Chunder N Chen C Umetsu SE Winandy S Wells AD. Ikaros enforces the costimulatory requirement for IL2 gene expression and is required for anergy induction in CD4^+^ T lymphocytes. J Immunol. (2007) 179:7305–15. 10.4049/jimmunol.179.11.7305 18025173

[106] Thomas RM Chen C Chunder N Ma L Taylor J Pearce EJ. Ikaros silences T-bet expression and interferon-gamma production during T helper 2 differentiation. J Biol Chem. (2010) 285:2545–53. 10.1074/jbc.M109.038794 19923223 PMC2807311

[107] Sehgal K Das R Zhang L Verma R Deng Y Kocoglu M. Clinical and pharmacodynamic analysis of pomalidomide dosing strategies in myeloma: impact of immune activation and cereblon targets. Blood (2015) 125:4042–51. 10.1182/blood-2014-11-611426 25869284 PMC4481593

[108] Kini Bailur J Mehta S Zhang L Neparidze N Parker T Bar N Changes in bone marrow innate lymphoid cell subsets in monoclonal gammopathy: target for IMiD therapy. Blood Adv. (2017) 1:2343–7. 10.1182/bloodadvances.2017012732 29296884 PMC5729633

[109] Klose CS Artis D. Innate lymphoid cells as regulators of immunity, inflammation and tissue homeostasis. Nat Immunol. (2016) 17:765–74. 10.1038/ni.3489 27328006

[110] Taylor S Huang Y Mallett G Stathopoulou C Felizardo TC Sun MA. PD-1 regulates KLRG1(+) group 2 innate lymphoid cells. J Exp Med. (2017) 214:1663–78. 10.1084/jem.20161653 28490441 PMC5461001

[111] Lesokhin AM Ansell SM Armand P Scott EC Halwani A Gutierrez M. Nivolumab in patients with relapsed or refractory hematologic malignancy: preliminary results of a phase Ib study. J Clin Oncol. (2016) 34:2698–704. 10.1200/JCO.2015.65.9789 27269947 PMC5019749

[112] Ribrag V Avigan DE Martinelli G Green DJ Wise-Draper T Posada JG Pembrolizumab monotherapy for relapsed/refractory multiple myeloma: phase 1b keynote-013 study. Haematologica (2017) 102:114.

[113] Schumacher TN Schreiber RD. Neoantigens in cancer immunotherapy. Science (2015) 348:69–74. 10.1126/science.aaa4971 25838375

[114] Mateos MV Hernandez MT Giraldo P De La Rubia J De Arriba F Corral LL. Lenalidomide plus dexamethasone versus observation in patients with high-risk smouldering multiple myeloma (QuiRedex): long-term follow-up of a randomised, controlled, phase 3 trial. Lancet Oncol. (2016) 17:1127–36. 10.1016/S1470-2045(16)30124-3 27402145

[115] Wilson L Cohen AD Weiss BM Vogl DT Garfall AL Capozzi DL Pembrolizumab in combination with pomalidomide and dexamethasone (PEMBRO/POM/DEX) for pomalidomide exposed relapsed or refractory multiple myeloma. Blood (2016) 128:2119.

[116] Badros A Hyjek E Ma N Lesokhin A Dogan A Rapoport AP. Pembrolizumab, pomalidomide, and low-dose dexamethasone for relapsed/refractory multiple myeloma. Blood (2017) 130:1189–97. 10.1182/blood-2017-03-775122 28461396

[117] Pawarode A D'Souza A Pasquini MC Johnson B Braun T Dhakal B Phase 2 study of pembrolizumab during lymphodepleted state after autologous hematopoietic cell transplantation in multiple myeloma patients. Blood (2017) 130:339.

[118] Thanendrarajan S Puryear J Schinke CD van Rhee F Zangari M Mathur P Nivolumab for treatment of advanced, refractory, high-risk multiple myeloma. Blood (2017) 130:1858.

[119] Biran N Andrews T Feinman R Vesole DH Richter JR Zenreich J A phase II trial of the anti -PD-1 monoclonal antibody pembrolizumab (MK-3475) + lenalidomide plus dexamethasone as post autologous stem cell transplant consolidation in patients with high-risk multiple myeloma. Blood (2017) 130:1831.

[120] Boddupalli CS Bar N Kadaveru K Krauthammer M Pornputtapong N Mai Z. Interlesional diversity of T cell receptors in melanoma with immune checkpoints enriched in tissue-resident memory T cells. JCI Insight (2016) 1:e88955. 10.1172/jci.insight.88955 28018970 PMC5161225

[121] Das R Bar N Ferreira M Newman AM Zhang L Bailur JK. Early B cell changes predict autoimmunity following combination immune checkpoint blockade. J Clin Invest. (2018) 128:715–20. 10.1172/JCI96798 29309048 PMC5785243

[122] Murillo O Arina A Hervas-Stubbs S Gupta A Mccluskey B Dubrot J. Therapeutic antitumor efficacy of anti-CD137 agonistic monoclonal antibody in mouse models of myeloma. Clin Cancer Res. (2008) 14:6895–906. 10.1158/1078-0432.CCR-08-0285 18980984 PMC2583963

[123] Westwood JA Matthews GM Shortt J Faulkner D Pegram HJ Duong CP. Combination anti-CD137 and anti-CD40 antibody therapy in murine myc-driven hematological cancers. Leuk Res. (2014) 38:948–54. 10.1016/j.leukres.2014.05.010 24934848

